# Identification of Hepatitis E Virus in the Feces of Red Foxes (*Vulpes vulpes*)

**DOI:** 10.3390/ani10101841

**Published:** 2020-10-10

**Authors:** Zsófia Lanszki, Kornélia Kurucz, Safia Zeghbib, Gábor Kemenesi, József Lanszki, Ferenc Jakab

**Affiliations:** 1 National Laboratory of Virology, Szentágothai Research Centre, University of Pécs, 7624 Pécs, Hungary; lanszkizsofi@gmail.com (Z.L.); kornelia.kurucz@gmail.com (K.K.); zeghbib.safia@gmail.com (S.Z.); kemenesi.gabor@gmail.com (G.K.); 2 Institute of Biology, Faculty of Sciences, University of Pécs, 7624 Pécs, Hungary; 3 Carnivore Ecology Research Group, Szent István University, 7400 Kaposvár, Hungary; lanszkij@gmail.com

**Keywords:** *Hepeviridae*, *Orthohepevirus C*, cvHEV, small mammal, zoonoses, taxonomy, diet

## Abstract

**Simple Summary:**

Orthohepeviruses, commonly known as Hepatitis E virus (HEV), is a diverse virus group belonging to the family of *Hepeviridae* and is responsible for acute hepatitis in humans worldwide. These viruses show a relatively strict host specificity, e.g., rodent-related, avian-related, or even bat-related virus groups. However, similar (HEV-like) viruses have been identified in carnivores; some of them form a new genetically separated group, while others show a close evolutionary relationship with the rodent-related group, thus makes the strict host-specificity questionable and the classification of these new strains uncertain. Herein, we investigated feces of red foxes, the most widespread carnivore species worldwide, to identify the Hepatitis E virus and to ascertain their evolutionary origin via sequencing. The non-invasively collected fecal samples can provide information about the presence of viruses specific to the host and viruses derived from their prey as well. The virus we detected from our samples showed a very close relationship (91% identity) with rodent-related HEV described before from common voles, whilst a more distant relationship (85%) with fox-specific HEV strains was observed. Our results strongly support “the dietary-origin” of unclassified HEV-like strains described from various predator species.

**Abstract:**

Orthohepeviruses (HEV) can infect a wide range of animals, showing a relatively strict host specificity; however, its zoonotic potential, natural transmission in the wildlife are less known. Several new HEV-like viruses have been identified in various animal species, including carnivores; however, the phylogenetic relationship among these viruses is poorly resolved, since some of them were known as rodent-related so far. The red fox, the most widespread carnivore worldwide, is a known reservoir of several viruses that transmit from wildlife to humans or domestic animals; they might have a defined role in the circulation of rodent-borne HEV. In this study, we performed a HEV survey by heminested RT-PCR (Reverse Transcription PCR) on red fox fecal samples to investigate the presence of HEV in red foxes living in natural conditions, and to explore the origin of the virus via phylogenetic analysis. Out of the 26 investigated samples, HEV RNA was identified in one sample. Following Sanger sequencing, the novel sequence displayed 91% identity on the nucleotide level with recently published European common vole-HEV derived from *Microtus arvalis*. In contrast, it shared 85% nucleotide similarity with HEV strains described previously in red foxes. Our results strongly support “the dietary-origin” of unclassified HEV-like strains described from predators that usually prey on rodents.

## 1. Introduction

Orthohepeviruses (known as Hepatitis E viruses (HEV), Family: *Hepeviridae*) show a relatively strict host specificity, as supported by the latest classification (ICTV), which was based on phylogeny and host range of these viruses [1]. Accordingly, *Orthohepevirus A* comprises sequences found in humans, causing acute hepatitis worldwide, in addition to other domestic animals and wild-living mammal species (pigs, wild boar, rabbit, deer, mongoose, and camel) (HEV1-8). *Orthohepevirus B* (Avian-HEV) and *Orthohepevirus D* (Bat-HEV) contain viruses of chickens and different bat species, while *Orthohepevirus C* encompasses sequences found in rodents (rats, mice, and voles) and, interestingly, in carnivores as the ferret, farmed mink or red fox [1,2,3,4]. So, in general, members of the *Orthohepevirus* genus infect a wide range of animals, although the exact host range of them remains obscure, primarily due to the discrete nature of HEV infections. HEV often presents undetectable pathology in infected organisms. Usually, viral load remains low and the viral shedding is prolonged or chronicle [5]. However, in the last decade, several new HEV-like viruses have been identified in a variety of animal species; their phylogenetic position is unclear. Therefore most of them are waiting for further classification.

Carnivore-derived HEV strains within the *Orthohepevirus C* species were increasingly discovered during the last decade. The first molecular evidence of a Carnivore-derived HEV was obtained from household pet ferrets (*Mustela putorius*) in the Netherlands [6]. A similar finding was described from farmed American minks (*Neovison vison*) in Denmark [7] and represented as a new HEV variant. Further evidence of Carnivore-HEV was mentioned from red foxes (*Vulpes vulpes*) in the Netherlands [8] and recently in Germany [9]. All of these putative novel variants clustered with Rat-HEV, which was the exclusively known group within *Orthohepevirus C* before, but clustered in a separate phylogenetic branch that was distinct from other previously described Rat-HEV variants. Multiple detections of these variants from different geographical locations may suggest these carnivores as the reservoirs of the virus [10]. Since most of these novel variants were found in fecal samples of these animals, the dietary origin of the identified viruses was assumed, considering that these carnivores generally consume rodents [11]. However, according to studies conducted on red foxes in Germany, the high prevalence of HEV antibodies among this animal population indicated an endemic infection [9].

From this perspective, the red fox is one of the most common and widespread members of the order Carnivora in the world [12]. It inhabits a wide variety of habitats, including natural or semi-natural areas (forests, wetlands, and grasslands), human-dominated agricultural areas, and settlements, including big cities [12,13]. It prefers small rodents, especially *Microtus* voles [14]. As a food generalist predator, besides small rodents, as primary foods, depending on habitat, eats lagomorphs, birds, insects, fruit garbage, and carrion of domestic animals or wild ungulates as well [15]. Consumption and control of pest rodents have economic importance. Still, its invasive appearance may have an adverse impact on smaller prey species, e.g., nesting birds or lagomorphs on natural or semi-natural habitats [16]. The home range size varies greatly (8–3420 ha) depending on the ecological conditions of the habitat [12]. Long-range forays and dispersal are rare events [17], but according to a study [18], the cumulative dispersal movements of individuals ranged from 132 to 1036 km. Foxes could be potential sentinel indicators in terms of their epidemiological appearance. Notable that foxes can be natural reservoir hosts of several pathogens, may be effective vectors of zoonotic diseases that pose an important risk for domestic animals and humans as well [19,20,21,22]. Therefore, the red fox is an ecologically and also epidemiologically important mesopredator [15].

This study was conducted to investigate the presence of Hepatitis E virus RNA in red foxes living in natural conditions, and to elaborate a phylogenetic analysis based on the resulting nucleotide sequence along with previously described sequences. Subsequently, we aimed to justify or refute the possible dietary origin of the novel rodent-related variants in carnivores.

## 2. Materials and Methods

### 2.1. Study Area and Sample Collection

The fecal samples of red foxes were collected in the Kis-Balaton area, within strictly protected marshlands (Western Hungary, the center of surveyed lines, N46.6711, E17.2250). The area is a Natura 2000 site and a Ramsar site and part of the Balaton Uplands National Park. In the marshland, the common reed (*Phragmites australis*) is dominant, but willows (*Salix* sp.) and poplars (*Populus* sp.) are located along the embankments passing through the area. The study area is situated in the continental climatic region, but there are some Mediterranean features (i.e., moderately warm and wet, and relatively mild winter). The Kis-Balaton is an important nesting, wintering, and migration area for birds and a favorable habitat for amphibians and numerous mammals. The most abundant carnivore is the red fox [23].

For our investigations, fresh and intact fecal samples of red foxes were collected, altogether four times between September and December 2018. Feces (each corresponding to one intact fecal sample) were collected on a 6.77 km long standard route (four lines, mean length 1.69 km/line) through the marshland on embankments and stored natively at −80 °C until laboratory processing. Samples were collected by ecologists who can distinguish feces of different Carnivora species based on position, special odor, size, and shape characteristics [23,24].

### 2.2. PCR Reaction and Sequencing

For molecular detection of HEV RNA, a heminested RT-PCR targeting the RdRp-encoding domain (RNA-dependent RNA polymerase gene) of the ORF1 of HEV was used, as described before Drexler, et al. [25]. Afterward, to obtain a more representative genome segment (846 bp) for phylogenetic reconstruction, we used further nested RT-PCR with primer sets (HEV-RdRp/F1—HEV-RdRp/R1 and HEV-RdRp/F2—HEV-RdRp/R2) published previously [26]. The detailed PCR experiments, sequencing, and phylogenetic analysis are similar to those described before [26]. Briefly, nested PCRs were performed with QIAGEN OneStep RT-PCR Kit (Qiagen, Hilder, Germany) and GoTaq G2 Flexi DNA Polymerase Kit (Promega, Madison, WI, USA) following the manufacturer’s recommendations. Final amplicons were sequenced bidirectionally with BigDye Terminator v1.1 Cycle Sequencing Kit according to the manufacturers’ protocol on ABI Prism 310 DNA Sequencer platform (Applied Biosystems, Foster City, CA, USA).

### 2.3. Phylogenetic Analysis

Before the phylogenetic inference, sequences of interest were retrieved from GenBank (NCBI, Bethesda, MD, USA) and aligned with our obtained sequence using the MUSCLE alignment tool (Drive5, Mill Valley, CA, USA). Then, MEGA X software (MEGA, Pennsylvania, PA, USA) was employed for the best substitution model selection. Subsequently, the phylogenetic tree was implemented using neighbor-joining method with the Kimura 2-parameter method and 1000 bootstrap in MEGA X [27]. Thus the resultant-tree was then edited in iTOL (iTOL, Heidelberg, Germany) [28].

### 2.4. Diet Composition Analysis

Diet composition analysis was performed from the feces of animal that has proven to be HEV-positive. The sample was prepared by a standard wet procedure [24]. The prey species were identified from undigested remains, as teeth using a Levenhuk 870T binocular microscope (Levenhuk, Tampa, FL, USA) [23].

## 3. Results

### 3.1. PCR Reaction and Sequencing

Altogether, *n* = 26 fox feces samples were included in the HEV RT-PCR screening. Hepatitis E virus was identified in one sample, collected at the beginning of December. Based on the GenBank BLASTn (NCBI, Bethesda, MD, USA) search, the resultant sequence (GenBank accession number: MN906015) showed (92%) identity with the European *Orthohepevirus C* strain (KU670940) identified previously in fecal samples collected from wild birds of prey, *Falco tinnunculus* (kestrel) in Hungary [29]. Besides, high nucleotide sequence similarity was observed with recently published European common vole-hepeviruses (cvHEV) described from *Microtus arvalis*. For instance, our sequence shared 91% identity with a Hungarian partial sequence (MH581173) [26] and 99% with both German and Czech Republic complete genomes (MK192406 and MK192408) [4]. Furthermore, it showed a lower nucleotide similarity (85%) to Fox Hepatitis E virus described previously in fecal samples of the red fox (KC692370) from the Netherlands [8].

### 3.2. Phylogenetic Analysis

**Figure 1:**
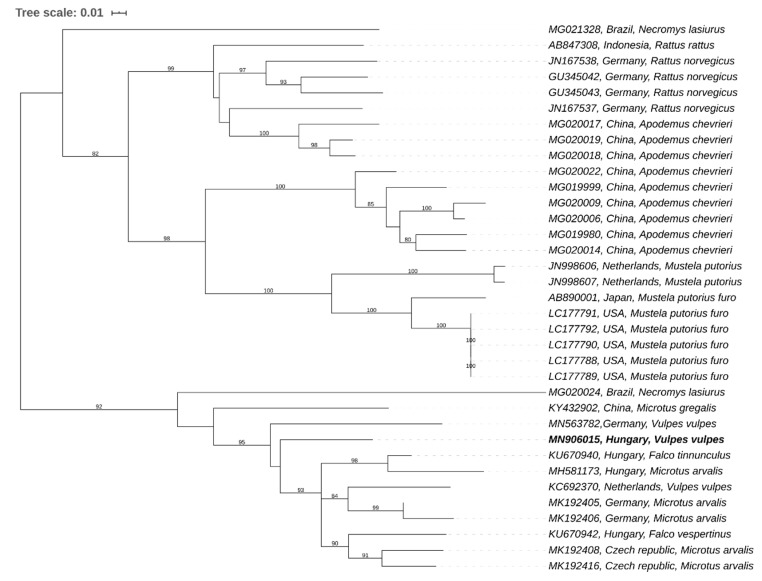
The evolutionary history of the partial RdRp nucleotide sequences was inferred using the Neighbor-Joining method. The optimal tree with the sum of branch length = 3.83355834 is shown. The confidence probability (multiplied by 100) that the interior branch length is greater than 0, as estimated using the bootstrap test (1000 replicates is shown next to the branches. The tree is drawn to scale, with branch lengths in the same units as those of the evolutionary distances used to infer the phylogenetic tree. The evolutionary distances were computed using the Kimura 2-parameter method and are in the units of the number of base substitutions per site. The rate variation among sites was modeled with a gamma distribution (shape parameter = 4). This analysis involved 36 nucleotide sequences. Codon positions included were 1st + 2nd + 3rd + Noncoding. All positions with less than 95% site coverage were eliminated, i.e., fewer than 5% alignment gaps, missing data, and ambiguous bases were allowed at any position (partial deletion option). There was a total of 271 positions in the final dataset. Evolutionary analyses were conducted in MEGA X. The sequence of interest is mentioned in bold.

The phylogenetic analysis supports the aforementioned results. Based on the phylogeny (Figure 1), our sample clustered with the cvHEV group, including the European common vole-hepeviruses rather than the novel HEV strain recently described from foxes in Germany (accession number: MN563782) [9]. Thus, suggesting that our sample contained cvHEV DNA originated from common voles (Figure 1).

### 3.3. Diet Composition

Based on the diet composition analysis, two rodent species (belong to Cricetidae: Arvicolinae) were determined from the feces of the HEV-positive red fox. These species were the common vole and European water vole (*Arvicola amphibius*). Remains of other foods did not present in the sample.

## 4. Discussion

Within the wide range of Hepatitis E viruses, *Orthohepevirus C* is known to be associated with rodents. Although, since it’s the first report in Europe from Norway rats (*Rattus norvegicus*) [30], several new Rat-HEV-like sequences have been described in different animal species other than rodents, including carnivores and birds of prey [3,4]. However, all of these new strains cluster to the rodent-associated *Orthohepevirus C*, their origin becomes questionable, as all of these novel variants were found in fecal samples, it might be more likely that the virus identified from predator animals derived originally from prey rodent species. Multiple detections of HEV strains in ferrets from different geographical locations (including North-America, Japan, and Europe) supported them as the most likely reservoir of this virus [10]. Since then, it has been proved that the virus causes acute hepatitis or induces a persistent infection in ferrets [31]. Furthermore, HEV strains originated from ferrets in Europe formed a distinct phylogenetic cluster from Rat-HEV, indicating that ferret-HEV is an individual strain within the *Orthohepevirus C*, classified as Carnivore-HEV (or genotype HEV-C2) and then referred all strains came from carnivores (i.e., red fox and American mink) to this group [3,10]. Meanwhile, strains originated from foxes and kestrels remained taxonomically unclassified [2].

The potential “dietary-origin hypothesis” has been discussed before. For instance, HEV RNA detected in common voles from Hungary showed a close genetic relationship to the previously described kestrel-derived HEV-strain from the same country, and to fox-derived strain from the Netherlands, supported the dietary-origin of strains detected from predators, while ferret-HEV strains segregated from them, suggesting that ferrets may carry a different virus strain than foxes or kestrels [26]. Based on their phylogenetic reconstruction, also a strict host species, specificity was supposed within the *Orthohepevirus C* species, since their common vole-derived sequences separated clearly from other Muridae-associated strains (rats and other *Apodemus* species) [26]. This assumption was later confirmed, at least partially by Ryll, et al. [4], who investigated the genomic and spatial variability of hepevirus in European common voles (cvHEV). Based on complete genome sequence analyses, they found high similarity and strong phylogenetic relationship between the cvHEV sequences (from Germany, Czech Republic, and Hungary) and the above mentioned kestrel-derived HEV strain from Hungary. As well, they confirmed that members of the species *Orthohepevirus C* are associated with rodents only and refuted an evolutionary origin of these viruses in avian hosts [4]. However, fox-derived hepevirus sequences were not involved in their analyses.

Our results are consistent and complementary to the abovementioned conclusions, as we found a strain very similar to cvHEV, in addition to the presence of common vole in the fox’s feces, which is native and common species of the investigated wetland area, and belong to the natural diet of red foxes [32]. Considering that only fecal samples were investigated and just a short screening fragment within the highly conserved coding region of the RdRp of HEV was determined, strong conclusions may not be drawn. Prpić, et al. [32] screened blood, spleen, and liver tissue samples of 50 red foxes in Croatia. However all samples were PCR negative for HEV RNA, it was not confirmed with serological investigations [32]. In contrast, a Syrian brown bear (*Ursus arctos syriacus*) in a German zoo was PCR positive for Rat-HEV without any disease symptoms, and serological evidence of Hepatitis E virus infection was confirmed in this animal, assumed a spillover infection of Rat-HEV from free-living Norway rats [3]. Recently, Eiden, et al. [9] performed serological studies, where the high antibody prevalence of the virus demonstrated endemic HEV infections within a fox population in Germany, furthermore HEV genome sequence was reported in one case, also clustered to the *Orthohepevirus C* group. Since it is unknown if HEV associated clinical signs in foxes, it is assumed that they can be reservoir hosts for the virus [9].

The knowledge about the pathomechanisms in wildlife and the zoonotic potential of these viruses are still poorly understood. Whereas Cricetidae-specific HEV genotype was only recently discovered, further investigations of rodent species belong to this taxa are needed to get more insights about this new genotype. The investigation of prevalence, genomic diversity and most importantly the infectivity of fox-derived HEV samples may reveal additional ecological and transmission aspects of Ortohepevirus genus. For a better exploration, more complex investigations would be necessary for the future, including serological studies, concurrent PCR tests for feces and serum, as well as traditional diet composition analysis [23] of infected animals. So far, among the Hepatitis E viruses, genotypes of *Othohepevirus A* species were associated with zoonotic potential and human disease, represent the most common cause of acute hepatitis in humans worldwide [3]. Besides that, serological evidence of infection with Rat-Hepatitis E virus or antigenically related agent (based on HEV-C1-specific IgG ELISA) was described in German forestry workers [33], and in hospitalized patients with febrile illness in Vietnam [34]. Furthermore, Rat-Hepatitis E virus was linked to severe acute hepatitis in immunocompetent patients in Canada [35] and liver transplant patients in China [36], highlighting the zoonotic potential of rodent-borne Hepatitis E viruses.

## 5. Conclusions

As the red fox is the most common wild-living carnivore, it is optimal for targeted assessments of pathogens. Although in our study, red fox feces were investigated, these non-invasively collected samples can provide information about the presence of known and unknown viruses specific for the host and viruses derived from their prey as well. That methodology could be applied more widely to survey the geographic distribution of viruses, without the necessity of invasive survey techniques, for example, live trapping or shooting animals. Although HEV was detected in one sample only in the examined natural habitat, it indicates the feasibility of fox samples as practical indicators for HEV presence in a particular area, and applicability of the fox as a sentinel for the detection and eventually monitoring of hepevirus in the environment.
